# Facepiece filtering respirators with exhalation valve should not be used in the community to limit SARS-CoV-2 diffusion

**DOI:** 10.1017/ice.2020.244

**Published:** 2020-05-15

**Authors:** Mariachiara Ippolito, Pasquale Iozzo, Cesare Gregoretti, Giacomo Grasselli, Andrea Cortegiani

**Affiliations:** 1 Division of Anaesthesia, Analgesia, Intensive Care and Emergency, Department of Surgical, Oncological and Oral Science, Policlinico Paolo Giaccone, University of Palermo, Italy; 2 Department of Anesthesia and Intensive Care, Policlinico Paolo Giaccone, University of Palermo, Palermo, Italy; 3 Dipartimento di Anestesia, Rianimazione ed Emergenza-Urgenza, Fondazione IRCCS Ca’ Granda Ospedale Maggiore Policlinico, Milan, Italy; 4 Department of Pathophysiology and Transplantation, University of Milan, Milan, Italy

*To the Editor*—From the first identified cases of COVID-19 onward, the pandemic spread of COVID-19 has presented difficult challenges for the scientific community. Many countries have already experienced periods of social lockdown, with the aim of containing the virus but with dramatic economic consequences. To balance health and economic and social needs in the long term, de-escalation of quarantine restrictions has been proposed in many countries.

During normal speech, a huge number of droplets are produced, and face covering may be effective in limiting the distance reached by the droplets, potentially reducing the transmission of the virus from individuals who are unaware that they are infected.^1^

Face covering with masks or tissue has been widely recommended as a complementary measure to reduce the infection rate in the community by limiting the excretion of droplets from asymptomatic or presymptomatic individuals.^2^ In this context, some governments are ordering face covering, especially during activities when social distancing is impossible or difficult (eg, using public transportation and visiting grocery stores or supermarkets, etc).^2,3^

Such measures should be intended as a protection towards the community and not as self-protection. A distorted comprehension of the real aim and a scarce knowledge of the differences among protective devices, has led many people to start using facepiece filtering respirators (FFRs) instead of the suggested nonmedical or medical masks, which are the most appropriate devices for source control, especially in the context of a pandemic.

FFRs are disposable filtering media, designed to provide the wearer an inward protection from inhaling contaminants conveyed by respiratory droplets or aerosols.^4^ On one hand, this ‘panic buying’ of FFRs may have contributed to the lack of supplies available for those employed in risky settings, such as healthcare workers frequently exposed to aerosol generating procedures, and it has also likely encourages counterfeiting.^5^ On the other hand, the uncontrolled sale of FFRs to people who are unaware of their specific features and are untrained in their use can create additional risks: incorrect doffing procedures can increase cross contamination; a false perception of safety can reduce the compliance to other measures (ie, hand hygiene, respiratory etiquette, social distancing); and even worse, the use of FFRs with exhalation valves in the community may be an additional and underrecognized transmission source.

The risks related to the presence of an exhalation valve are not intuitive for the general population and should not be silenced by institutions and governments. FFRs endowed with exhalation valves are meant for prolonged use, such as during extended work shifts when the wearer may experience discomfort and heat due to high resistance during exhalation. The valve opens only during the expiration, lowering resistance encountered during expiration. At lower inward pressures than those created by the expiratory airflow, the valve closes and, despite minimal inward leakage, filtering occurs during inspiration, together with a more comfortable expiration.^6^

The functioning of exhalation valves poses major concerns about outward protection, which is reasonably diminished by FFRs. Several institutions have already expressed concerns about their use outside the recommended context. The European Centres for Disease Prevention and Control (ECDC) and Africa Centre for Disease Prevention and Control have provided clear statements against their use in the community setting.^7,8^ The US Centers for Disease Prevention and Control (CDC) recommended against their use in healthcare settings where a sterile field must be maintained, thus implying that the outward protection is not provided by FFRs.^9^ Recently, the City and County of San Francisco explicitly listed respirators with one-way valves among those forbidden for use in the community, clarifying that they ‘allow droplets out of the mask, putting others nearby at risk,’ thus not complying with the face-covering order.^10^

Communication campaigns should aim to promote the wearing of masks as a source control measure and to increase awareness that FFR supplies are already insufficient to protect highly exposed workers. Indeed, institutions and governments should consider preventing free marketing of FFR with valves, given that their indiscriminate use in the community setting can determine an additional and under tracked risk for the population.
